# Risk Communication on Zoonoses and Antimicrobial Resistance—How Do Exotic Pet Owners Perceive the Communication of Their Veterinarians?

**DOI:** 10.3390/ani14142035

**Published:** 2024-07-10

**Authors:** Amelie Lisa Arnecke, Stefan Schwarz, Antina Lübke-Becker, Katharina Charlotte Jensen, Christina Herre, Mahtab Bahramsoltani

**Affiliations:** 1 Institute of Veterinary Anatomy, School of Veterinary Medicine, Freie Universität Berlin, Koserstraße 20, 14195 Berlin, Germany; christina.herre@fu-berlin.de (C.H.); mahtab.bahramsoltani@fu-berlin.de (M.B.); 2 Institute of Microbiology and Epizootics, School of Veterinary Medicine, Freie Universität Berlin, Robert-von-Ostertag-Straße 7, 14163 Berlin, Germany; stefan.schwarz@fu-berlin.de (S.S.); antina.luebke-becker@fu-berlin.de (A.L.-B.); 3 Veterinary Centre for Resistance Research (TZR), School of Veterinary Medicine, Freie Universität Berlin, Robert-von-Ostertag-Straße 8, 14163 Berlin, Germany; 4 Institute for Veterinary Epidemiology and Biostatistics, School of Veterinary Medicine, Freie Universität Berlin, Königsweg 67, 14163 Berlin, Germany; charlotte.jensen@fu-berlin.de

**Keywords:** risk communication, risk perception, risk awareness, zoonoses, antimicrobial resistance (AMR), pathogen transmission, one health, exotic pets

## Abstract

**Simple Summary:**

Exotic animals can carry pathogens that may spread to humans and other animals. When traded and kept as pets, the risk of disease transmission increases as there is a higher potential of close contact and stress involved that can weaken the animals’ immune system, making them more likely to shed pathogens. This becomes especially important for households with children younger than five years, elderly and pregnant people or immunocompromised individuals. Thus, this survey investigated how exotic pets are kept, the advice given by veterinarians and how well veterinarians communicate the risks of zoonoses and antimicrobial resistance. The results showed that owning exotic pets comes with several health risks. However, pet owners generally felt satisfied with their veterinarians’ communication, especially when they had consulted a veterinarian for a longer time. Despite this, pet owners expressed a desire for more frequent information on these topics. Therefore, it is important to provide more educational resources. Enhancing education and training for veterinarians, particularly in universities, could improve their communication with pet owners about the risks associated with exotic pets. This study highlights the important role of veterinarians in the prevention of pathogen transmission from animals to humans through targeted risk communication.

**Abstract:**

Exotic animals traded and kept as pets can transmit a variety of diseases to humans and other animals, and vice versa. Therefore, it is essential for pet owners, particularly vulnerable groups, to be informed about associated risks. Veterinarians play a crucial role in informing pet owners about health risks associated with zoonotic pathogens and antimicrobial resistance (AMR) and should, therefore, have good communication skills to effectively transfer information to pet owners. Thus, exotic pet owners in Germany were surveyed on animal husbandry, veterinary consultation and risk communication. To evaluate the perception of communication, a self-developed questionnaire was used to derive a communication score. The perception of veterinarian communication received a high average score showing a high level of satisfaction. The duration of the veterinarian–client relationship was associated with better communication perception, and the frequency of communication on zoonoses and AMR was associated with the presence of a permanent veterinarian. However, the results indicated that the frequency of disseminated information on zoonoses and/or AMR from veterinarians was lower than desired by the pet owners. Therefore, more educational material on zoonoses and AMR should be made available, and the awareness concerning risk communication should be increased by further education and training at universities.

## 1. Introduction

Over the last decades, there has been an emergence of more than 100 different human diseases originating from wild animals, posing significant concerns for human health [1]. In this context, the live animal trade, including the exotic pet trade, was identified as one of five major contributors to the spread of pathogens within the wildlife trade [2]. Zoonotic pathogens are relevant for both humans and animals as they can be transmitted between and among them, leading to illness and fatalities, posing a threat to public health, veterinary health and wildlife populations [3,4,5].

Private pet ownership, including exotic pet animals, is widespread in Germany and is particularly common among families with children [6,7]. Contact with pets is linked to people’s mental and physical wellbeing [8,9]. However, contact with exotic pets especially is associated with specific risks, as they can transmit a variety of diseases to humans [4,10,11,12,13,14]. In addition, bacterial pathogens exhibiting antimicrobial resistance (AMR) pose a significant health threat by reducing the effectiveness of antimicrobial agents in treating infections [15]. Previous studies support the theory on potential cross-transmission of antimicrobial-resistant bacteria between animals and humans [16,17,18,19,20,21,22]. As antimicrobial-resistant pathogens are found in small and exotic animals, pets can be considered as potential reservoirs [23,24,25]. Consequently, there is a high likelihood of resistant bacteria transferring between pets and humans [26].

This undermines efforts to control infectious diseases in both humans and animals, emphasizing the interconnectedness of health across different species and ecosystems [27]. Due to the close interaction between humans and animals, it is vital to acknowledge the different routes of infection transmission but also to be aware of the individual risks connected to vulnerable groups, such as children younger than five years, adults older than 65 years, people with weakened immune systems and pregnant people [28]. Besides this, the population of immunocompromised individuals has experienced a steep exponential rise over the past few decades [29].

Thus, the role of medical professionals is crucial to translate risk communication messages into clinical practice [30]. However, previous findings have revealed that physicians tend to consider veterinarians as having a superior knowledge and understanding of zoonoses [31,32]. Studies suggest that veterinarians have indeed a superior comprehension of zoonotic pathogens and demonstrate higher proficiency in diagnosing zoonoses when compared to physicians [33]. As veterinarians hold specialized knowledge and expertise in animal health but also have a unique position at the human–animal interface, they bear a distinct responsibility to the public health sector [34]. In contrast to physicians, veterinarians are required to have a broad understanding of many species, which could lead to gaps in specialized knowledge. Furthermore, their expertise with wildlife species and exotic pets is often limited due to the focus on common domestic animals in primary training and practice. Specialisations require additional postgraduate training beyond the scope of the standard veterinary education [35,36,37]. Nevertheless, veterinarian communication plays a significant role strengthening the bond between veterinarians and pet owners by amplifying the clients’ willingness to follow recommendations given by the veterinarian [38]. However, low compliance with deworming protocols and gaps in pets’ immunization indicate a noticeable decrease in the utilization of preventive medicine among pet owners, thereby prompting the need for improved communication and information strategies [39,40,41]. Furthermore, emerging zoonoses are unpredictable events that require special communication strategies. This issue should be addressed and practiced in training programs, so that the veterinary community is prepared for an outbreak situation [42]. Previous studies have shown that veterinary practitioners can further benefit from training on specific communication skills [43]. Research supports the idea that strong communication skills, particularly in veterinary science and related health sectors are vital [44]. Despite this, veterinarians face a distinct challenge in interacting and communicating effectively with pet owners [45,46], and the perception of risk depends not only on the objective reality but also on the people’s needs, goals, past experiences, expectations and reference points [47]. Thus, it is important to assess pet owners on their perception of veterinarian communication in order to adapt the risk communication strategies [48].

The aim of this study was to gain insights into exotic pet owners’ perception of veterinarian communication on zoonoses and AMR. In particular, the frequency of risk communication and the perception of communication in relation to risk factors and risk behaviours should be assessed. Furthermore, the association between risk communication and the veterinarian–client relationship as well as the reason for the consultation should be determined. Thus, the findings can provide important insights for practicing veterinarians, since they have an important role in risk communication to strengthen global health at the human–animal interface.

## 2. Participants, Materials and Methods

### 2.1. Participants

For this study, exotic pet owners in Germany were surveyed. The survey, conducted via Lime-Survey Cloud Version 5.6.56^®^ from December 2022 to April 2023, was promoted on social media (Facebook and Instagram). Participants could access the survey by using a link or scanning a QR code. The participation was voluntary, and participants were given the freedom to withdraw from the study at any point without facing consequences. Before starting the survey, participants had to read the legal notice and agree to our privacy policy. In addition, they had to confirm that they are at least 18 years old. The study concept was approved by the Ethics Committee (ZEA-Nr. 2022-018) of the Freie Universität Berlin.

### 2.2. Questionnaire

The questionnaire consisted of six sections. Every participant was queried on risk factors, their pet(s) and associated risk behaviour, the veterinarian–client relationship and if/why veterinarian consultation took place within the past 12 months. A distinction was made between routine (routine visit or preventive check-up, e.g., immunisation, blood, or dental control) and specific (specific symptoms and pathogen detection). If they stated having visited the veterinarian, they were asked if communication on zoonoses and/or AMR took place. If so, they were asked for their perception of the communication with veterinarians on zoonoses and/or AMR. If not, they proceeded directly to the section containing the final questions on veterinary advice and needs. To avoid misunderstandings or misinterpretations, all technical terms were explained beforehand and repeated in the description section of the specific question. The definitions of zoonoses and AMR were based on the definition of the World Health Organisation [15,49]. Furthermore, the classification of exotic pets (bird, fish, mammal and terrarium animal including reptile, amphibian, insect and arachnid) was based on the data from the Zentralverband Zoologischer Fachbetriebe Deutschlands e.V. (ZZF) [6].

#### 2.2.1. Risk Factors

The questions on risk factors gathered information on vulnerable groups in the participants household, including immunocompromised individuals, children under six years, elderly people over 65 years and pregnant individuals (Table 1). The variable ‘children under six’ was created, combining the variables ‘infant’ and ‘toddler’. The variable ‘immunocompromised individuals’ was created by adding the variables ‘cancer, chronically ill, diabetes and immunosuppressed’.

#### 2.2.2. Pet(s) and Associated Risk Behaviours

The second section of the questionnaire focused on information related to the respondent’s pet(s) (animal classes/species) and risk behaviours connected to the origin (wild-caught and/or import from abroad), access to shared living spaces and risky feeding practices (raw meat/fish, offal, uncooked bones, frozen and/or live prey) (Table 2).

#### 2.2.3. Veterinarian–Client Relationship

The section on the veterinarian–client relationship assessed the permanency, duration of the respondents’ relationship with their veterinarians and the satisfaction of care. If participants indicated that they did not have a permanent veterinarian, they were inquired about the reasons behind this decision. Information was assessed using the following questions (Table 3).

#### 2.2.4. Veterinary Consultation

In the veterinary consultation section, participants were asked if and why they visited the veterinarian during the past 12 months (Table 4). Regarding the reasons for the consultation, a distinction was made between routine visits including preventive check-ups (e.g., immunisation, blood or dental control) and consultations due to specific symptoms with pathogen detection. The variable ‘specific’ was combined from the different variables of the question assessing specific symptoms (gastrointestinal, urinary, skin, respiratory and others). Furthermore, the detection of pathogens was assessed using a list of the most relevant pathogens (Supplement S1), which was compiled through the literature research and in dialogue with practicing veterinarians. The skin and gastrointestinal tract have been identified as the most significant areas for zoonotic concerns [4,29,42,43]. Conversely, concerns about AMR are often linked to surgery but also to chronic conditions associated with various organ tracts, such as the gastrointestinal, urinary, skin and respiratory system [50,51,52].

#### 2.2.5. Veterinarian Communication

##### 2.2.5.1. Frequency of Veterinarian Communication

If participants selected that they visited the veterinarian for routine or specific reasons, they were asked whether communication on zoonoses and/or AMR took place (Table 5).

##### 2.2.5.2. Perception of Veterinarian Communication

A questionnaire was developed and validated to measure the perception of veterinarian communication by pet owners.

Development of the Questionnaire

The generated items relate to Friedemann Schulz von Thun’s four-sides model. This communication model is based on the assumption that every message always has four facets, which are referred to as the sides of the message, i.e., factual level, appeal level, relationship level and self-disclosure. The factual level indicates the information provided. The facet of the message that gives an indication of what the sender of the message wants the recipient to do is called the appeal level. The relationship level is the facet of the message that shows what the sender of the message thinks of the recipient and the relationship between them. The fourth facet of the message, self-disclosure, contains the aspects that the sender discloses about him- or herself in the message [53]. Four items were formulated for each side of the communication, which were assessed by the participants on a 4-point Likert scale ranging from 1 (disagree), 2 (rather disagree), 3 (rather agree) to 4 (agree).In addition, they had the option to choose ‘no answer’. The items regarding the factual level were aimed at whether the relevant information was given and whether it was formulated in a way that the pet owner could understand. To assess the communication at appeal level, the items related to whether appeals were made and explained in a comprehensive manner and if consequences for complying or not complying with the appeals were pointed out. Regarding the relationship level, the items were intended to determine the pet owners’ perception of the veterinarian’s appreciation, trust and empathy. For the assessment of self-disclosure, items were formulated to find out whether the veterinarian’s self-presentation led pet owners to perceive the veterinarian as professionally competent (Table 6).

Validation of the Questionnaire

To assess the validity of the self-developed questionnaire structure, a factor analysis based on the four-sides model was conducted, incorporating four items corresponding to each side. This analysis aimed at determining if the four sides would be an adequate instrument to assess communication in our questionnaire. Sensitivity controls revealed that the sides were not distinct: Cronbach’s Alpha was 0.891 for the 16 items indicating a high internal consistency. In addition, an exploratory factor analysis did not allow a distinction of the four sides into four factors. The Kaiser–Meyer–Olkin Measure of Sampling Adequacy of 0.567 and Bartlett’s test of sphericity (*p* < 0.001) already indicated a high correlation of communication variables. The resulting structure also pointed towards a one-factor solution with all items except the item ‘The veterinarian provided me with info material’ depicting high loadings upon the first factor extracted. As the Eigenvalue and Scree Plot also indicated a one-factor solution, the expected structure of the four sides could not be confirmed and was rejected. Therefore, it was decided to calculate a one-dimensional score from the mean value to represent the perceived communication.

#### 2.2.6. Veterinary Advice and Needs

The final chapter focused on the respondent’s final thoughts regarding veterinary advice. It assessed the respondent’s interest and needs for specific advice or guidance in the following areas: zoonoses, AMR, nutrition, behaviour, husbandry, immunisation, hygiene measurements and the treatment of endo- and ectoparasites (Table 7).

### 2.3. Statistical Analysis

Data were exported in Microsoft Excel 2024^®^, and the statistical analyses were carried out with IBM SPSS Statistics Version 29^®^. Only participants who finished the questionnaire were included in the analyses. Descriptive analyses were carried out.

#### 2.3.1. Analysis of Communication Frequencies

To gain an understanding of risk communication regarding zoonoses and AMR, we examined whether communication occurs and whether risk factors (the existence of vulnerable groups in the household and pathogen detection) and risk behaviours (feeding of raw meat/fish, offal, uncooked bones, frozen and/or live prey, free access to shared living spaces, imported and wild-caught animals) have an impact on the frequency. When correlating two categorical variables, a Chi^2^-Test was applied. If the assumption of having an expected count of at least 5 within each cell was violated, Fisher’s exact test was used.

#### 2.3.2. Analysis of Communication Perception

The mean score of the specific communication (specific symptom with pathogen detection) was compared with that of the routine communication (routine consultation and preventive health check-up) using the Mann–Whitney-U-Test. For the analysis, a distinction was not made between pathogens with zoonotic potential and those with multidrug-resistant potential; instead, the cases of confirmed pathogens were aggregated and compared with the communication during routine visits. The differences in communication (mean scores) to the duration of veterinary care were tested by calculating the Kruskal–Wallis-Test. For the pairwise comparison of groups (<1 year, 1–3 years, 3–6 years, >6 years), *p*-values were adjusted for multiple testing (Bonferroni correction). Besides that, the differences (mean scores) regarding risk factors and behaviours were tested. For all inferential analyses, a significance level of 5% was chosen.

## 3. Results

A total of 547 exotic pet owners participated. Out of those, 344 completed the survey.

### 3.1. Presence of Risk Factors in the Household

**Figure 1:**
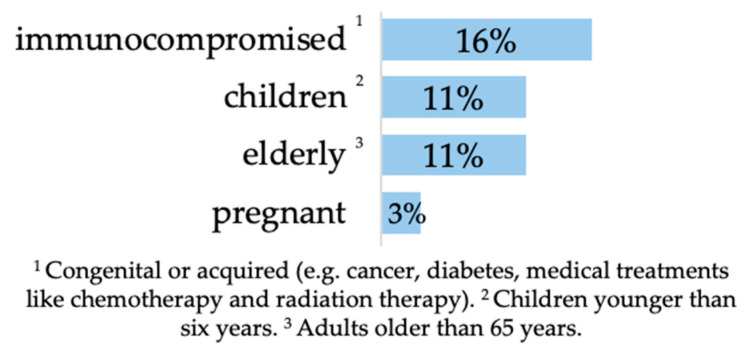
Presence of risk factors (vulnerable groups) in the household (immunocompromised *n* = 56, children *n* = 37, elderly *n* = 36, pregnant *n* = 10 of 344) multiple answers were possible.

Among the participants, almost one-third (32%, *n* = 111 of 344) stated having individuals from at least one vulnerable group in their household, including immunocompromised individuals, children younger than six years, elderly and pregnant people (Figure 1).

### 3.2. Pet(s) and Associated Risk Behaviours

Participants reported owning a variety of different exotic animals (reptiles 58%, exotic mammals 33%, birds 20%, insects 15%, arachnids 13%, fishes 13%, amphibians 8%, others 4%).

**Figure 2:**
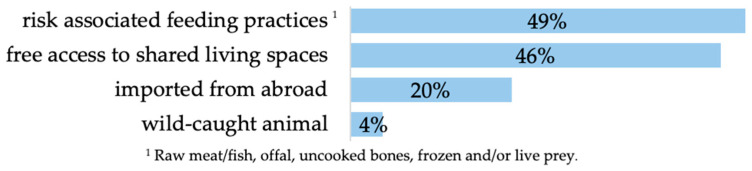
Presence of risk behaviour (risk-associated feeding practice *n* = 161 of 327, free access to shared living spaces *n* = 154 of 333, imported from abroad *n* = 64 of 326, wild-caught animal *n* = 12 of 344; multiple answers were possible).

Furthermore, 80% (*n* = 276 of 344) of the participants reported engaging in activities that could pose an increased risk of pathogen transmission (Figure 2). Nearly half of all participants fed raw meat/fish, offal, uncooked bones and frozen and/or live prey and allowed free access to shared living spaces. Besides this, about one quarter of the participants stated that at least one of their animals was imported from abroad, while a concerning 4% (*n* = 12 of 344) acknowledged owning a wild-caught animal.

### 3.3. Veterinarian–Client Relationship

**Figure 3:**
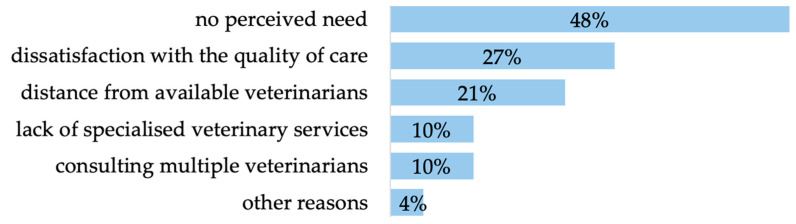
Reasons for not having a permanent veterinarian (no perceived need *n* = 25, dissatisfaction with the quality of care *n* = 14, distance from available veterinarians *n* = 11, lack of specialised veterinary services *n* = 5, consulting multiple veterinarians *n* = 5, other reasons *n* = 2 of 52; multiple answers were possible).

The results show that 85% (*n* = 283 of 335) of the participants had a permanent veterinarian (11% <1 year, 27% 1–3 years, 20% 3–6 years, 42% >6 years). A notable level of customer satisfaction is indicated by the fact that 74% (*n* = 208 of 281) chose ‘agree’ for the item ‘Are you satisfied with the care you receive from your veterinarian?’. The reasons for not having a permanent veterinarian (16%, *n* = 52 of 335) were no perceived need, dissatisfaction with the quality of care, distance from available veterinarians, lack of specialised veterinary services, consulting multiple veterinarians and other reasons (Figure 3).

### 3.4. Reasons for Veterinary Consultation

Within the past 12 months, 66% (*n* = 227 of 343) of the participants consulted a veterinarian. Among those participants who stated having visited a veterinarian, 64% (*n* = 146 of 227) did so due to a routine visit or preventive check-up, while 48% (*n* = 109 of 227) consulted the veterinarian due to specific symptoms. Out of these consultations, 38% (*n* = 87) were only due to specific symptoms, 22% (*n* = 50) were only for routine consultations, and 26% (*n* = 59) involved both specific symptoms and routine consultations.

The primary concerns prompting veterinary consultation revolved around the respiratory tract, followed by the skin, gastrointestinal and urinary tract. Additionally, they cited other symptoms of concern such as neurological, dental and ocular.

Among the participants who visited a veterinarian within the past 12 months, 11% (*n* = 25 of 227) of the participants reported the detection of pathogens with zoonotic (9%, *n* = 20 of 227) or/and AMR (4%, *n* = 9 of 227) potential. A total of 43 cases—including bacteria, fungi, protozoa, ectoparasites and endoparasites—were reported.

### 3.5. Veterinarian Communication on Zoonoses and AMR

#### 3.5.1. Frequencies of Veterinarian Communication

The frequency of communication on zoonoses and AMR was associated with the presence of a permanent veterinarian (*p* = 0.002). Among participants who consulted the veterinarian within the past 12 months, 20% (*n* = 46 of 227) reported receiving information on zoonoses and 17% (*n* = 39 of 227) on AMR. An association of the existence of vulnerable groups in the household (*p* = 0.454) or with other risk behaviours was not proven (*p* = 0.292).

Information on zoonoses was statistically significantly more frequently given (*p* < 0.001) during consultations due to specific reasons (50%, *n* = 25 of 50) compared to those who visited a veterinarian for routine reasons (22%, *n* = 31 of 144). Furthermore, during consultations prompted by specific reasons, pet owners received statistically more frequently (*p* < 0.001) information on zoonoses (50%, *n* = 25 of 50) than on AMR (18%, *n* = 24 of 132). During routine consultations, pet owners received information on zoonoses (22%, *n* = 31 of 144) with a similar frequency to AMR (17%, *n* = 24 of 144).

#### 3.5.2. Participant’s Perception of Veterinarian Communication

In general, the perception of veterinarian communication received a high average score of 3.7 out of a maximum 4.0 (mean; *n* = 72), most choosing ‘rather agree’ and ‘agree’ for the given items (Table 8). The second item of the relationship level, ‘I had the feeling that the veterinarian took my questions serious’, and the third item of the self-disclosure received ‘agree’ most frequently. The only exception was the fourth item of the appeal level: ‘The veterinarian provided me with informational material’, which received a high frequency of ‘disagree’, showing that informational material was distributed in less than half of the cases.

##### Perception of Communication Connected to Risk Factors and Risk Behaviours

The presence of vulnerable groups in the household resulted in a higher average communication score of 3.8 (*n* = 19) in comparison to households with no vulnerable groups of 3.6 (*n* = 53). This result was not statistically significant, but a trend could be detected (*p* = 0.052).

The presence of risk behaviour resulted in a slightly higher average score of 3.7 (*n* = 59) in comparison to households with no risk behaviours of 3.6 (*n* = 12), with no statistically significant difference (*p* = 0.635).

##### Perception of Communication Connected to Duration of Veterinary Care

**Figure 4:**
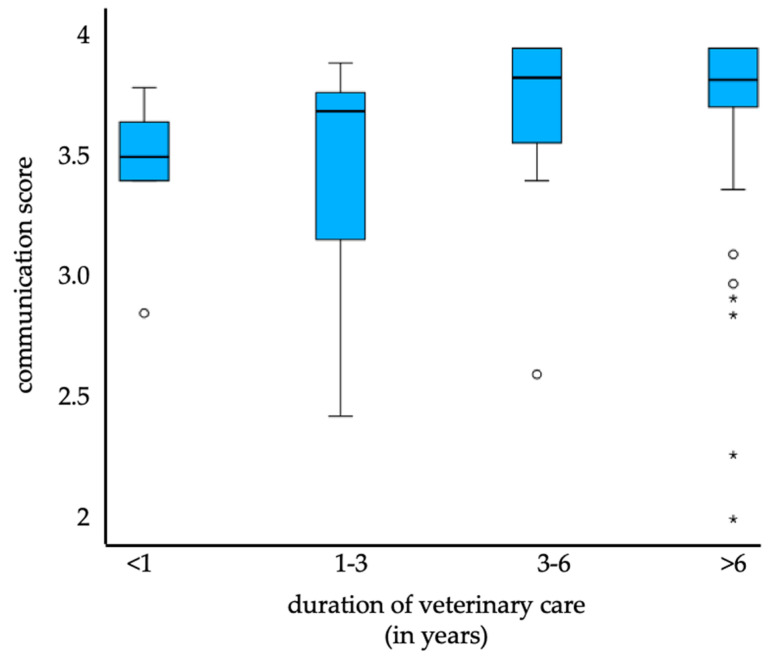
Average communication perception in relation to the duration of veterinary care (under one year: mean = 3.5, *n* = 5; one to two years: mean = 3.4, *n* = 7; three to six years: mean = 3.7, *n* = 16; over six years: mean = 3.7, *n* = 44; o = outlier, * = extreme value).

Among all participants who answered the communication model and stated having a permanent veterinarian, the following observation was made: the longer the pet owners were clients, the better they rated the communication (under one year: mean = 3.5, *n* = 5; one to two years: mean = 3.4, *n* = 7; three to six years: mean = 3.7, *n* = 16; over six years: mean = 3.7, *n* = 44) (Figure 4). This result was not statistically significant, but a trend could be detected (*p* = 0.057). Post hoc tests revealed that there was an improvement in perceived communication for pet owners being clients for three to six years compared to those who were clients for less than one year (z= −1.869, *p* = 0.062) and for those being clients for more than six years compared to the ones who were clients for one to three years (z= −2.213, *p* = 0.027).

##### Perception of Communication Connected to Reason of Consultation

The perceived communication during a routine visit revealed a slightly higher average score (mean = 3.7, *n* = 52) than the specific communication (mean = 3.6, *n* = 20), with no statistically significant difference (*p* = 0.113). The average score was slightly lower when a pathogen was detected (mean = 3.6, *n* = 31) compared to no detected pathogen (mean = 3.7, *n* = 41) but was not statistically significant (*p* = 0.103). Furthermore, the communication of zoonoses (mean = 3.7, *n* = 42) perceived a slightly higher average score than AMR (mean = 3.6, *n* = 30) but was not statistically significant (*p* = 0.707).

### 3.6. Veterinary Advice and Needs

**Figure 5:**
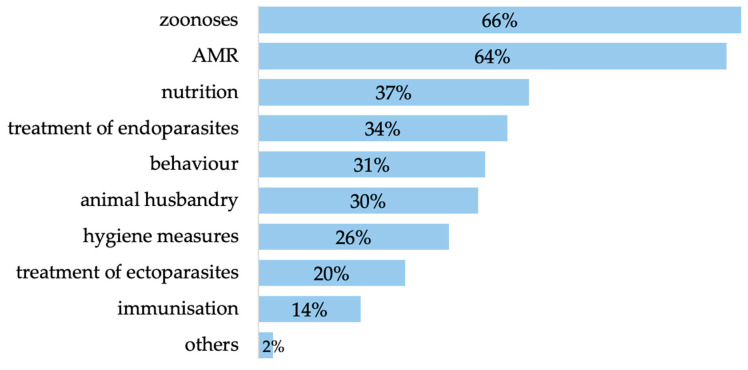
Topics for veterinary advice (zoonoses *n* = 195 of 296, AMR *n* = 193 of 301, nutrition *n* = 126, treatment of endoparasites *n* = 116, behaviour *n* = 105, animal husbandry *n* = 103, hygiene measures *n* = 90, treatment of ectoparasites *n* = 69, immunisation *n* = 48, others *n* = 7 of 344; multiple answers possible).

More than half (66%, *n* = 195 of 296) of the participants indicated that they would like veterinary advice on zoonoses and on AMR (64%, *n* = 193 of 301). Besides this, pet owners also wanted the veterinarian to provide information on nutrition, behaviour, animal husbandry, immunisation, treatment of endoparasites and ectoparasites, hygiene measures and other topics (Figure 5).

## 4. Discussion

Since the husbandry of exotic pets can lead to infections with zoonotic and antimicrobial-resistant pathogens [16,17,18,19,20,21,22,23,24,25], it is an essential task of veterinarians to provide pet owners with information about these risks [34]. The veterinarians’ communication skills as well as their knowledge and awareness play a significant role in the extent to which this information is distributed to and understood by pet owners and whether the veterinarians’ instructions are followed [38]. Therefore, the aims of this study were to assess (i) the role of veterinary practitioners in communicating risks of zoonoses and AMR as well as (ii) exotic pet owners’ perception of their veterinarian communication.

Previous studies have shown the presence of zoonotic and antimicrobial-resistant pathogens in exotic pets [4,10,11,12,13,14]. In the present study, 11% of the pet owners, who had consulted a veterinarian within the past 12 months, reported the detection of pathogens with zoonotic (9%) or/and AMR (4%) potential in their pet(s).

Cohort and case–control studies have indicated that certain groups—young children (under six years), older adults (65 years and older), immunocompromised individuals and pregnant individuals—are at a higher risk for zoonotic diseases. As their immune system is either impaired or not fully developed yet, these groups have an increased risk of falling ill and may experience more severe symptoms or disease progression compared to other patients [28,32,54,55,56]. Among the participants, 32% reported having individuals from such vulnerable groups in their household. In addition, 80% of the participants reported activities that could pose an increased risk of pathogen transmission. Nearly half of all participants reported feeding raw meat, fish, offal, bones and frozen and/or live prey. Investigations of various raw meat feeding approaches have validated the presence of pathogens, including antimicrobial-resistant pathogens, capable of causing severe human illnesses, and pets consuming raw meat may harbour these pathogens without exhibiting symptoms, serving as carriers and potential sources of transmission [57,58]. Especially vulnerable groups, who are more likely than others to fall seriously ill, face an elevated risk handling such products [59,60]. Besides this, about one quarter of the participants stated that at least one of their animals was imported from abroad, while, concerningly, 4% acknowledged owning a wild-caught animal. Those animals may pose a health risk to humans and other animals due to their potential to carry specific pathogens [61,62,63,64]. The stress of capture and transport, combined with a lack of strict health regulations, increases the risk of introducing new diseases and antibiotic-resistant bacteria into local ecosystems [65,66].

Therefore, a tailored risk communication is required for raising awareness and potentially preventing pathogen transmission [67,68,69,70]. The results show that the frequency of communication on zoonoses and AMR during veterinary consultation was higher when a pathogen was detected as during routine visits and was associated with the presence of a permanent veterinarian. Even though the overall perception of veterinarian communication received a high average score showing a high level of satisfaction, also the duration of the veterinarian–client relationship was positively associated with a better communication perception. These findings align well with previous studies, as they also underscore the significant role of the veterinarian–client relationship for successful communication [31], resulting in higher compliance and thus improved patient outcomes [49].

The results indicate a gap between the wish to be informed on zoonoses (66%) or AMR (64%) and the frequency of disseminated information from veterinarians to pet owners, as 20% of the participants who consulted the veterinarian within the past 12 months received information on zoonoses and 17% on AMR. The gap between the wish to be informed and the actual frequency of information distributed to the pet owners might be connected to the fact that COVID-19 has raised awareness about zoonoses, connected health risks and the importance of preventive measures [71,72,73]. In addition, the veterinary community has several concerns, including the clients’ intent to adhere, the perception that adherence is redundant, misunderstanding or denial of risks, costs, rural culture, fear for reputation and the emotional state of the client [42]. These could be reasons why there is a gap between the good perception of veterinarian communication and the limited frequency of risk communication. However, it is important to pet owners that they receive the desired information in the necessary scope, in understandable language and ideally in several formats [74]. However, the pet owners surveyed in this study assessed the veterinarians’ communication regarding explanations of the cause and transmission of infections slightly, although not significantly, worse after pathogen detection compared to no pathogen detection. In addition, pet owners rarely reported having received information material on the pet’s infection. Additional information media, such as flyers that can be taken and read at home at leisure, could be a helpful alternative for people of distinct ages and academic backgrounds [74]. Therefore, increased efforts should be made to produce information material on zoonoses and AMR, tailored to pet owners and their pets in order to support veterinarians in the important task of maintaining the health of animals and humans. In addition, increasing the communication skills of veterinarians contributes to the effective education of pet owners about zoonoses and AMR. Thus, future veterinarians could significantly benefit from learning communication skills at a university level, not just for conveying complex medical information to pet owners, fostering trust and ensuring the compliance of pet owners with treatment plans but also to collaborate more efficiently with colleagues and other professionals in the public health sector [43,75,76,77].

Future studies and research could focus on assessing the effectiveness of information materials on zoonoses and AMR, understanding the factors influencing veterinarian selection and evaluating pet owners’ feeding practices. These areas could help to build a more detailed understanding of the veterinarian–client dynamic, ultimately improving pet care and health outcomes. Additionally investigating the impact of communication techniques of veterinarians and physicians and conducting longitudinal studies on pets’ and their owners’ health could provide valuable insights for improving not only veterinary care but also moving in the direction towards One Health.

There are some limitations connected to participant engagement in this study. The distribution method via the internet posed a barrier for some individuals, potentially leading to selection bias. Moreover, those persons who accessed the internet and were part of the groups where the survey was promoted and read the invitation might differ from the target population, as participation needs time, a general willingness to participate in surveys and the impression that the topic of the survey is appealing. Even though we limited the timeframe to 12 months, memory bias remains a potential limitation, as respondents might inaccurately recall their consultations or base their responses on more recent experiences or emotional states, thereby reducing the reliability and validity of the collected data. Another limitation factor is that veterinary consultations are stressful, which is why the message sent is not always the message received. Furthermore, the use of a 4-point Likert scale in the communication perception questionnaire may have provided too few gradations to yield distinct results, suggesting that the questionnaire should be retested with a Likert scale that has more gradations.

## 5. Conclusions

This survey of pet owners has shown that risk factors and risk behaviour in relation to zoonoses and AMR are of particular relevance to the husbandry of exotic pets. Veterinarians play a crucial role in communicating risks ofzoonoses and AMR associated with exotic pets to their owners. The results of this study have revealed that the duration of the veterinarian–client relationship contributes both to a higher frequency of communication on zoonoses and AMR and to pet owners’ perceptions of better veterinarian communication. However, since pet owners would prefer to receive significantly more information on zoonoses and AMR, more target-group-specific information materials should be made available to veterinarians for distribution to their clients. Here, the awareness of veterinarians should be increased by further education and the implementation of communication teaching during veterinary education at universities.

## Figures and Tables

**Table 1:** **Questions on risk factors (vulnerable groups) in the household.** (multiple answers possible: 1. and 2.).

1. Which age groups of people live in your household?
(infant ^1^, toddler ^2^, school-aged children, teenagers, adults, elderly ^3^)
2. Do any individuals of these groups live in your household?
(pregnant, cancer, chronical ill, diabetes, immunosuppressed ^4^)

^1^ under two years, ^2^ two to five years, ^3^ over 65 years, ^4^ congenital or acquired (e.g., medical treatments like chemotherapy and radiation therapy).

**Table 2:** **Questions on the pet(s) and associated risk behaviours.** (multiple answers possible: 1. and 2.).

1. Which type of pet(s) do you currently have, or have kept in the last year? (reptile, amphibian, bird, fish, arachnid, insect, exotic mammal, others)
2. Which is the origin of your pet?
(breeder, private purchase, pet fair, internet portal, animal shelter or organisation, garden centre or pet store, wild-caught, others)
3. Is your pet imported from abroad?
(yes, no)
4. Does your pet have access to shared living spaces?
(yes, no)
5. Do you feed raw meat/fish, fresh offal, uncooked bones, frozen and/or live prey?
(yes, no)

**Table 3:** **Questions on veterinarian–client relationship.** (multiple answers possible: 4.).

1. Do you have a permanent veterinarian?
(yes, no)
if yes: 2. How long have you been a regular client of your veterinarian?
(<1 year, 1–3 years, 3–6 years, >6 years)
3. Are you satisfied with the care you receive from your veterinarian?
(disagree, rather disagree, rather agree, agree)
if no: 4. Which is the reason why you do not have a permanent veterinarian?
(no need, multiple, dissatisfaction with quality, distance, others)

**Table 4:** **Questions on veterinary consultation.** (multiple answers possible: 2., 3., 5.).

1. Have you visited a veterinarian within the past 12 months?
(yes, no)
if yes: 2. For which reason have you consulted the veterinarian?
(routine check-up, acute or chronic illness, emergency, surgery, others)
3. Does or did your pet have symptoms?
(gastrointestinal, skin, respiratory, urinary, others)
4. Was a pathogen detected?
(yes, no)
if yes: 5. The following pathogen(s) was/were detected during the examination.
(Supplement S1)

**Table 5:** **Questions on communication frequencies.** (multiple answers possible: 3.).

1. The veterinarian spoke with me in this context about zoonoses.
(yes, no)
2. The veterinarian spoke with me in this context about AMR.
(yes, no)
3. The veterinarian spoke with me about following pathogen(s).
(Supplement S1)

**Table 6:** **Items on pet owners’ perception of veterinarian** **communication.**

Side of the Message [53]	Items (Specific)	Items (Routine)
factual level	The veterinarian explained the cause and transmission of my pet’s infection (e.g., pathogen characteristics).	The veterinarian explained the cause and transmission of zoonoses/AMR (e.g., pathogen characteristics).
factual level	I was able to follow the veterinarian’s explanations on the cause and transmission of my pet’s infection (e.g., language, choice of words, pace of speech).	I was able to follow the veterinarian’s explanations on the cause and transmission of zoonoses/AMR (e.g., language, choice of words, pace of speech).
factual level	The veterinarian’s explanations of the cause and transmission of my pet’s infection were formulated in a language I could understand.	The veterinarian’s explanations of the cause and transmission of zoonoses/AMR were formulated in a language I could understand.
factual level	The veterinarian emphasised the most important points about the cause and transmission of my pet’s infection (e.g., transmission mechanism, pathogen characteristics).	The veterinarian emphasised the most important points on the cause and transmission of zoonoses/AMR (e.g., transmission mechanism, pathogen characteristics).
appeal level	The veterinarian provided me with instructions to prevent the transmission of my pet’s infection (e.g., cleaning and disinfecting hands, food bowls, toys, pet beds, baskets, blankets and pet toilets).	The veterinarian provided me with instructions to prevent the transmission of zoonoses/AMR (e.g., cleaning and disinfecting hands, food bowls, toys, pet beds, baskets, blankets and pet toilets).
appeal level	The veterinarian explained the reasons for the recommended behavioural instructions connected to my pet’s infection (e.g., preventing the transmission of the pathogen to me, maintaining the infection and reinfection of the pet).	The veterinarian explained the reasons for the recommended behavioural instructions connected to zoonoses/AMR (e.g., preventing the transmission of the pathogen to me, maintaining the infection and reinfection of the pet).
appeal level	The veterinarian explained to me the advantages of the behavioural instructions connected to my pet’s infection (e.g., prevention of new outbreaks of disease, health care and health protection, cost savings).	The veterinarian explained to me the advantages of the behavioural instructions connected to zoonoses/AMR (e.g., prevention of new outbreaks of disease, health care and health protection, cost savings).
appeal level	The veterinarian provided me with informational material on my pet’s infection (e.g., information sheets, magazines, websites).	The veterinarian provided me with informational material on zoonoses/AMR (e.g., information sheets, magazines, websites).
relationship level	The veterinarian demonstrated understanding towards me during the conversation on my pet’s infection.	The veterinarian demonstrated understanding towards me during the conversation on zoonoses/AMR.
relationship level	I had the feeling that the veterinarian took my questions serious during the conversation on my pet’s infection.	I had the feeling that the veterinarian took my questions serious during the conversation on zoonoses/AMR.
relationship level	The veterinarian was able to understand what the behavioural instructions, connected to my pet’s infection, mean to me.	The veterinarian was able to understand what the behavioural instructions, connected to zoonoses/AMR, mean to me.
relationship level	I trust the veterinarian’s advice connected to my pet’s infection.	I trust the veterinarian’s advice connected to zoonoses/AMR.
self-disclosure	I felt that the veterinarian gave me competent advice connected to my pet’s infection.	I felt that the veterinarian gave me competent advice connected to zoonoses/AMR.
self-disclosure	I had the feeling that the conversation with the veterinarian on my pet’s infection, took place at eye level.	I had the feeling that the conversation with the veterinarian on zoonoses/AMR took place at eye level.
self-disclosure	I had the feeling that I could ask the veterinarian questions about my pet’s infection at any time.	I had the feeling that I could ask the veterinarian questions about zoonoses/AMR at any time.
self-disclosure	I had the feeling that it was important to the veterinarian that I understand the causes and consequences of my pet’s infection.	I had the feeling that it was important to the veterinarian that I understand the causes and consequences of zoonoses/AMR.

**Table 7:** **Questions on veterinary advice and needs.** (multiple answers possible: 3.).

1. Would you like to receive veterinary advice on zoonoses?
(yes, no)
2. Would you like to receive veterinary advice on AMR?
(yes, no)
3. Would you like to receive veterinary advice on any of the following topics:
(nutrition, behaviour, husbandry, immunisation, treatment of endoparasites and application of deworming medication, treatment of ectoparasites and application of tick and flea control products, hygiene measurements)

**Table 8:** **Communication perception.** Connected to the four levels of communication (factual level, appeal level, relationship level, self-disclosure) based on Schulz von Thun’s four-sides model. Four items were formulated for each level of the communication, which were assessed by the participants on a 4-point Likert scale ranging from 1 (disagree), 2 (rather disagree), 3 (rather agree) to 4 (agree).

Item	Perception (Specific)	Perception (Routine)
factual level:	missing/disagree/rather disagree/rather agree/agree *n* = 20	missing/disagree/rather disagree/rather agree/agree *n* = 52
The veterinarian explained the cause and transmission.	1/1/2/6/10	0/0/4/8/40
I was able to follow the veterinarian’s explanations.	3/0/0/3/14	1/0/0/7/44
The veterinarian’s explanations were formulated in a language I could understand.	3/0/1/3/13	0/0/2/6/43
The veterinarian emphasises the most important points about the cause and transmission	5/1/1/5/8	1/0/3/5/43
appeal level:		
The veterinarian provided me with instructions to prevent the transmission.	4/2/1/1/12	1/1/2/7/41
The veterinarian explained the reasons for the recommended behavioural instructions	3/1/1/1/14	1/1/2/6/42
The veterinarian explained me the advantages of the behavioural instructions.	4/1/1/2/12	2/1/4/6/39
The veterinarian provided me with information material.	4/11/2/0/3	0/24/4/5/19
relationship level:		
The veterinarian demonstrates understanding towards me.	2/0/0/5/13	4/0/0/6/42
I had the feeling that the veterinarian took my questions serious.	1/0/0/2/17	0/0/0/2/50
The veterinarian was able to understand what the behavioural instructions mean to me.	4/0/0/4/12	6/0/0/8/38
I trust the veterinarian’s advice.	2/0/0/3/15	0/0/0/4/48
self-disclosure:		
I felt that the veterinarian gave me competent advice.	2/0/1/2/15	0/1/2/3/46
I had the feeling that the conversation took place at eye level.	0/0/0/4/16	0/0/1/3/48
I had the feeling that I could ask the veterinarian questions at any time.	0/0/0/2/18	0/0/1/1/50
I had the feeling that it was important to the veterinarian that I could understand the causes and consequences.	4/0/0/2/14	2/0/2/4/44

## Data Availability

Data are contained within the article or Supplementary Materials.

## Supplementary Materials

The following supporting information can be downloaded at: https://www.mdpi.com/article/10.3390/ani14142035/s1, Supplement S1: List of Pathogens; Supplement S2: Raw Data.
