# Supplementary material for: Risk Communication on Zoonoses and Antimicrobial Resistance—How Do Exotic Pet Owners Perceive the Communication of Their Veterinarians?
Source: Animals (Basel). 2024 Jul 10;14(14):2035. doi: 10.3390/ani14142035 (PMC11273538; doi:10.3390/ani14142035)
Supplement: Supplementary file 1 [file animals-14-02035-s001.zip › Arnecke et al. 2024_Supplement S1_List of Pathogens.pdf]

List of Pathogens

Acinetobacter (*Acinetobacter baumannii*)
Campylobacter (*Campylobacter* spp., *Campylobacter jejuni*)
Clostridia (*Clostridioides difficile*)
Coliform bacteria (*Escherichia coli*)
Cryptosporidium (*Cryptosporidium* spp.)
Dermatophytes (*Trichophyton mentagrophytes*, *Microsporum canis*)
Giardia (*Giardia* spp., *Giardia intestinalis*)
Klebsiella (*Klebsiella* spp.)
Mites (*Histiella trombidiiformis, Ophionyssus natricis*, *Trombiculida* spp., *Sarcoptes scabiei*)
Mycobacteria (*Mycobacterium* spp., *M. chelonae, M. kansasii, M. marinum, M. szulgai, M. terrae, M. ulcerans*)
Pseudomonads (*Pseudomonas aeruginosa*)
Pinworms (*Oxyurid* sp.)
Rabies (Rabies Virus, RABV)
Salmonella (*Salmonella* spp., S. Typhimurium, S. Enteritidis)
Staphylococcus (*Staphylococcus* spp., *S. aureus, S. pseudintermedius*, Methicillin-resistant *S. aureus* (MRSA), Methicillin-resistant *S. pseudintermedius* (MRSP))
Toxoplasma (*Toxoplasma gondii*)
others[1]

[1]free-text field with comment option
